# Use of scientific evidence by dentists in Brazil: Room for improving the evidence-based practice

**DOI:** 10.1371/journal.pone.0203284

**Published:** 2018-09-19

**Authors:** Ana Paula R. Gonçalves, Marcos B. Correa, Flavia P. S. Nahsan, Carlos J. Soares, Rafael R. Moraes

**Affiliations:** 1 Graduate Program in Dentistry, Federal University of Pelotas, Pelotas, RS, Brazil; 2 Graduate Program in Dentistry, Federal University of Sergipe, Aracaju, SE, Brazil; 3 Graduate Program in Dentistry, Federal University of Uberlândia, Uberlândia, MG, Brazil; Eberhard-Karls-Universitat Tubingen Medizinische Fakultat, GERMANY

## Abstract

This study investigated the use of scientific evidence and the practice of evidence-based dentistry (EBD) among dentists working in Brazil. An online questionnaire was emailed to dentists registered with Brazilian state dental councils. The questionnaire assessed the demographic, educational, and work characteristics of the sample, along with habits in reading scientific articles and other updating resources. Data were analyzed descriptively and by using Bonferroni, Kruskal-Wallis, ANOVA, and T-test statistical methods (α = 0.05). A total of 795 responses were received between June, 2015 and January, 2016. The response rate was not calculated because it was not possible to determine precisely how many dentists received these e-mails. Over 77% of the responding dentists completed postgraduate training. They referred mainly to books, scientific or clinical journals, conferences, and short-term courses for updating their knowledge. Dentists who reported having a habit of reading scientific journals (60.9%) showed a preference for reading case reports, clinical research articles, and literature reviews. Most dentists (77.5%) reported changing their clinical behaviors or procedures based on information gleaned from journal articles. The types of articles that led them to change their clinical practices were primarily clinical research articles and case reports. Working in the public sector was also associated with a lower prevalence of a habit of reading scientific journals and practicing EBD (i.e., self-reported practice). The results suggest that dentists are interested in reading journal articles, especially those addressing clinical outcomes, and that there is room for improving the practice of EBD, particularly in the public sector.

## Introduction

The concept of “evidence-based health care” emerged in the late 1990s, and refers to the use of the best available evidence in clinical decision-making to increase the quality and predictability of treatment [1,2]. Accordingly, evidence-based dentistry (EBD) is the practice of referring to available data in the literature on oral health care while also considering the complex environment in which clinical decisions are made. The American Dental Association defines EBD as “an approach to oral healthcare that requires the judicious integration of systematic assessments of clinically relevant scientific evidence, relating to the patient’s oral and medical condition and history, with the dentist’s clinical expertise and the patient’s treatment needs and preferences.” This implies considering, in addition to specific patient information, relevant data from the literature, and then translating these data into clinical decisions [3].

The routine implementation of EBD should begin in dental undergraduate education through training in biostatistics and critical reading of scientific literature [4]. Dentists who lack experience in reading articles might face challenges in interpreting certain terminology [5], particularly when the article is in a foreign language [6]. Other potential obstacles are personal motivations and financial issues originating in either the professional or patient, which might cause them to choose one intervention or treatment over another [7,8]; an intense workload [9,10]; or even an excess of publications in dentistry [7,11,12]. In addition, studies that report conflicting results might also end up confusing professionals [10]. These obstacles might lead dentists to make decisions based on their previous experiences or opinions from colleagues rather than on current scientific evidence [7].

Studies carried out in many countries have assessed professional and knowledge updating and information-seeking practices among dentists [9,10,13–16]. In these studies [9,10,13–16], factors such as a lack of clear answers to clinical questions, insufficient computer literacy, and exposure to studies reporting diverging outcomes were mentioned as barriers to accessing information and should be addressed to overcome the problems of transferring information into practice. Most of those studies also indicated a low reliance on evidence-based information resources during clinical practice, and that dentists’ experience plays a significant role in their updating patterns. One recent study with dental practitioners showed that those who are older and did not attend continuing education courses are less likely to use the most up-to-date clinical techniques [17]. This finding highlights that the communication of scientific evidence to dentists and the dissemination of EBD are goals for researchers to pursue in order to improve oral health care. This study aimed to analyze, via an electronic survey, the practices related to searching and using up-to-date scientific dental information among dentists working in Brazil. The study hypothesis was that the practice of EBD would be influenced by academic experience and work variables of the dentists including their time in practice, postgraduate education, and work sector.

## Materials and methods

### Questionnaire development

This study was approved by the Ethics Research Committee, Federal University of Pelotas, Brazil (protocol 1.085.285). In this cross-sectional study, an online questionnaire asking about dentists’ professional updating practices was sent to dentists working in Brazil. In a pilot study, the self-administered questionnaire was sent by e-mail to a sample of 20 graduate students to pretest it in terms of wording, sequence, and internal consistency, and to assess the content validity, i.e. if the questions were considered relevant to analyze the updating and EBD practices of the respondents. These aspects were not evaluated quantitatively, but the questions were edited based on the pilot study until a consensus on content validity was reached.

### Subject recruitment

The final questionnaire was hosted online in Google Forms and comprised up to 37 items (27 categorical, 7 visual analogue scales and 3 open-ended questions), depending on the combination of responses. This report does not address all the content from this questionnaire. All dentists must be registered to a regional dental council to work legally in Brazil, thus we invited all Brazilian state dental councils to cooperate with the study by emailing the survey to all dentists registered with those councils. The cover e-mail invited the dentist to respond to the survey, presented the purpose of the questionnaire and the time estimated to answer it (15 min), introduced the researchers responsible for the study, and clearly mentioned that there would be no identification of the respondent in any form. The first page of the survey reinforced some aspects already presented in the e-mail, including the purpose of the study and confidentiality of the data, and additionally provided information on multiple choice questions and how the results of the research would be made available in the literature: one Master dissertation and one scientific article. The e-mail was sent directly to the personal addresses present in the records of the regional dental councils. The dentist was required to read the e-mail, click on the link to the survey, and agree to participate (first question) in order to access the remainder questionnaire. Hence, no informed consent form was required. The e-mails were sent between June and December 2015, and responses were received up to January 2016.

### Survey content

The questions presented in the questionnaire were based on previous studies that have conducted similar surveys in dentistry [9–11,13,15]. The questions were divided into four main groups: 9 questions with the general characteristics of the sample (e.g., gender, age, education, and current professional practices); graduate courses completed and current or past experience with educational activities (15 questions); informational resources and methods most commonly used for professional updating (7 questions); and use of scientific evidence in clinical practice (5 questions). For the general characteristics, the variables of interest were gender, region, and population size of the largest city where the dentist had worked (up to 50,000; from 50,001 to 300,000, or above 300,000 inhabitants). Time in practice was recorded as number of years completed and later categorized as “up to 5 years,” “from 6 to 15 years,” or “more than 15 years.” The dentists were also asked about whether they had completed postgraduate education, whether they were attending continuing education at the moment, and their dental specialty and work sector (public, private, or teaching activities).

Regarding the information resources for professional updating, different options were presented to the dentists (in the form of yes/no questions). They were also asked about the frequency of use of resources that they reported using. Regarding scientific journals, dentists were asked about their habits of reading journals, preferred journal origin (national/international), and the type of articles they typically read. An open question allowed them to name the journals that they most commonly read. The participants were also asked whether they had made changes to any clinical practices or procedures based on information published in scientific journals; if they responded in the negative, they were asked the reason for not changing. A visual analogue scale (VAS) ranging from 0 (never) to 10 (always) was used to assess how often dentists inferred that the information presented in scientific journals did not match the reality of clinical practice. Dentists completed a similar VAS to indicate how much of their own clinical practice was based on information from scientific journals. While it is challenging to assess practice of EBD, we nevertheless believe that this latter VAS could serve as a useful self-perceived measure.

### Data analysis

Descriptive statistics were calculated to identify the absolute and relative frequencies of categorical variables and the distributions of the numerical variables. The association of the dichotomous outcomes with the categorical independent variables was analyzed using the chi-square test. The association between categorical variables and numerical outcomes was assessed using either t-test or analysis of variance (with Bonferroni-corrected post hoc testing). The Kruskal-Wallis test was used for numerical outcomes with non-normal distributions. Significance was set at α = 0.05, and all analyses were carried out using Stata 11.0 (StataCorp, College Station, TX, USA).

## Results

### Characteristics of gender, age, education, and professional practice of the responding dentists

We received 795 responses, which represents about 0.3% of the dentists registered in the Brazilian dental councils [18]. However, the response rates were not calculated because it was not possible to determine precisely how many dentists received or accessed the e-mails. Although the records in all state dental councils include the dentist e-mail as a necessary information, some e-mail addresses could be outdated if the dentists did not provide updated information to the council recently. Table 1 shows the absolute and relative frequencies for the demographic, education, and work characteristics of the responding dentists. The sample comprised mostly woman (56.5%), and their average age was 38 ± 11 years. The majority worked in the Southeast region of Brazil (49.6%) and in cities with a population over 300,000 inhabitants (52.6%). There was a balance between the groups of professional experience, with 29.2% of dentists having up to 5 years of experience, 34% having between 6 and 15 years, and 36.9% having over 15 years. Over 77% of the dentists had completed postgraduate training and 16.1% held an MSc or PhD. The specialties included general dentistry (23.6%), orthodontics (15.5%), restorative dentistry (9.2%), public oral health (9.1%), prosthodontics (8.9%), and endodontics (8.9%). The main work position was private practice (77.1%). A total of 13.2% of the respondents were involved in teaching activities.

**Table 1:** Demographic, education, and work characteristics of the respondents, Brazil, 2017 (N = 795).

Variable/Category	n	
**Gender**		
Female	446	56.5%
Male	344	43.5%
**City population**		
Up to 50,000	164	20.6%
Between 50,001 and 300,000	213	26.8%
Above 300,000	418	52.6%
**Professional experience (time in practice)**		
Up to 5 years	232	29.2%
Between 6 and 15 years	270	34.0%
More than 15 years	293	36.9%
**Postgraduate education (completed)**		
None	178	22.4%
Residency or advanced specialty training	489	61.5%
MSc or PhD	128	16.1%
**Dental specialty**		
General dentistry	188	23.6%
Orthodontics	123	15.5%
Restorative dentistry	73	9.2%
Dental public health	72	9.1%
Prosthodontics	71	8.9%
Endodontics	71	8.9%
Pediatric dentistry	45	5.7%
Implantology	43	5.4%
Oral and maxillofacial surgery	31	3.9%
Periodontics	28	3.5%
**Work sector**		
*Public practice*		
Yes	331	41.6%
No	464	58.4%
*Private practice*		
Yes	613	77.1%
No	182	22.9%
*Teaching activities*		
Yes	105	13.2%
No	690	86.8%
**Currently attending continuing education**		
Yes	289	36.3%
No	506	63.7%

### Professional updating and information resources

**Fig 1:**
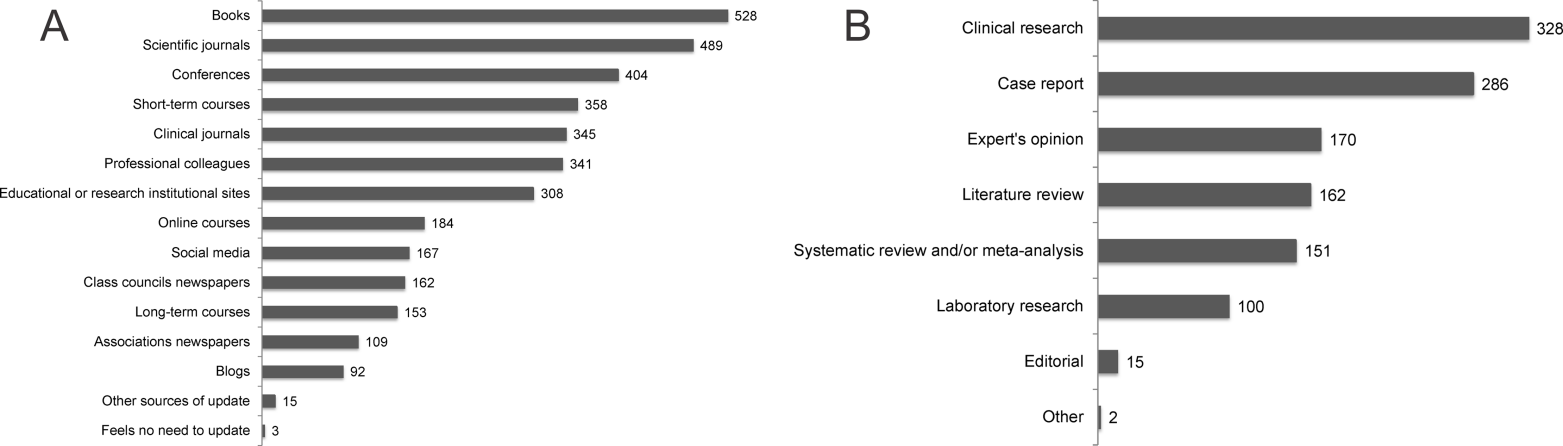
**Informational resources used by dentists for updating (A) and article types that most often made the dentists change their clinical behavior or procedures (B).** Scientific journals: journals that publish mainly original research articles; clinical journals: journals that publish mainly case reports.

Participants reported referring mainly to books, scientific or clinical journals, conferences, and short-term courses when they felt the need to update or deepen their knowledge (Fig 1A). Contact with colleagues (dentists) was also mentioned as a common source of updating. Websites, online courses, social media, and blogs were mentioned less often. When asked about the frequency of updating, 45% of respondents reported seeking information monthly or irregularly when they felt it necessary, 33% reported to seek for information weekly and 21% daily. Only 1% reported not seeking for information.

### Use of scientific evidence in practice

The dentists who reported having a habit of reading scientific journals (60.9% of the sample) showed a preference for reading case reports, clinical research articles, and literature reviews (Table 2). For the VAS asking how often they inferred that the information presented in the scientific journals did not match the reality of clinical practice, their mean score was 4.7 ± 2.4. Most dentists (77.5%) reported changing clinical practices or procedures based on information from journal articles. The types of articles that most often made dentists change their clinical practice were clinical research articles and case reports (Fig 1B). Of the 179 respondents who answered that they had never changed any clinical behavior or procedure based on scientific articles (Table 3), the main reason was not having a habit of reading these journals (40.2%). For the VAS asking the extent to which their clinical practice was based on information from scientific journals (Table 4), which was an indication of practicing EBD, the mean score (6.1±2.5) indicated that this extent was not substantial. In fact, 39.6% respondents had values ≤5, which could mean that information published in journals has a relatively minor influence on clinical decisions.

**Table 2:** Absolute and relative frequencies of article types in which the dentists claimed to search information, Brazil, 2017 (N = 484*).

Variable	Article type, n (%)	
	Yes	No
Case report	371 (76.6%)	113 (23.4%)
Clinical research	351 (72.5%)	133 (27.5%)
Literature review	297 (61.4%)	187 (38.6%)
Systematic review	179 (37.0%)	305 (63.0%)
Laboratorial research	167 (34.5%)	317 (65.5%)
Epidemiological research	164 (33.9%)	320 (66.1%)
Experts’ opinion	150 (31.0%)	334 (69.0%)
Editorial	67 (13.8%)	417 (86.2%)
Other	7 (1.4%)	477 (98.6%)

*Sample of dentists who claimed to seek information in scientific journals.

**Table 3:** Reasons that dentists do not change their clinical practices based on information published in journals, Brazil, 2017 (N = 179*).

Variable	n	
Do not have a habit of reading scientific articles	72	40.3%
Believe that articles do not reflect the clinical reality	36	20.1%
Important information is published in a foreign language	17	9.5%
Do not feel it is necessary	16	8.9%
Use other resources to change clinical practices	10	5.6%
Believe more information is required beforehand	9	5.0%
Other reasons	19	10.6%
Do not believe the information published in scientific journals	-	-

*Sample of respondents who claimed to have never changed clinical practices or procedures based on information from scientific articles.

**Table 4:** Associations of education and work variables with the extent to which dentists indicated that their practice was based on scientific evidence, Brazil, 2017 (n = 795).

Variable/Category	Mean (SD)	P-value
**Professional experience (time in practice)**		
Up to 5 years	6.6 (2.4) A	
Between 6 and 15 years	6.0 (2.5) B	0.05*
More than 15 years	5.7 (2.6) B	0.001*
**Postgraduate education (completed)**		
None	5.6 (2.7) B	
Residency or advanced specialty training	5.9 (4.5) B	
MSc or PhD	7.4 (2.1) A	<0.001**
**Work sector**		
*Public practice*		
Yes	5.8 (2.5) B	0.004***
No	6.3 (2.5) A	
*Private practice*		
Yes	6.1 (2.5) A	0.784***
No	6.0 (2.6) A	
*Teaching activities*		
Yes	7.3 (2.1) A	<0.001***
No	5.9 (2.5) B	
**City population**		
Up to 50,000	5.9 (2.5) A	0.22****
Between 50,001 and 300,000	5.9 (2.6) A	
Above 300,000	6.2 (2.5) A	
**Currently attending continuing education**		
Yes	6.5 (2.5) A	<0.001***
No	5.8 (2.5) B	

*Bonferroni test

**Kruskal-Wallis test

***t-test

****ANOVA.

Distinct letters indicate significant differences between the categories within the same variable.

### Variables associated with reading journals and practicing EBD

Table 4 shows the results of the associations of the extent to which dentists based their practice on scientific evidence with the education and work variables. The main factors associated with greater EBD practice were having less professional experience (up to 5 years), holding an MSc or PhD, engaging in teaching activities, and currently attending continuing education. Working in the public sector was associated with reduced scores. Table 5 shows the results of the associations of having a habit of reading journals with the education and work variables. The factors associated with having a habit of reading journals were essentially the same as those associated with increased practice of EBD: holding an MSc or PhD, engaging in teaching activities, working in more populated cities, and currently attending continuing education. Public practice was associated with a lower prevalence of the habit of reading scientific journals.

**Table 5:** Associations of education and work variables with the habit of reading journals, Brazil, 2017.

Variable/Category	Read scientific journals, n (%)		P-value
	Yes, 484 (60.9%)	No, 311 (39.1%)	
**Professional experience (time in practice)**			
Up to 5 years	149 (64.2%)	83 (35.8%)	0.350
Between 6 and 15 years	165 (61.1%)	105 (38.9%)	
More than 15 years	170 (58.0%)	123 (42.0%)	
**Postgraduate education (completed)**			
None	90 (50.6%)	88 (49.4%)	<0.001
Residency or advanced specialty training	269 (55.0%)	220 (45.0%)	
MSc or PhD	125 (97.7%)	3 (2.3%)	
**Work sector**			
*Public practice*			
Yes	179 (54.1%)	152 (45.9%)	0.001
No	305 (65.7%)	159 (34.3%)	
*Private practice*			
Yes	374 (61.0%)	239 (39.0%)	0.890
No	110 (60.4%)	72 (39.6%)	
*Teaching activities*			
Yes	96 (91.4%)	9 (8.6%)	<0.001
No	388 (56.2%)	302 (43.8%)	
**City population**			
Up to 50,000	84 (51.2%)	80 (48.8%)	0.016
Between 50,001 and 300,000	137 (64.3%)	76 (35.7%)	
Above 300,000	263 (62.9%)	155 (37.1%)	
**Currently attending continuing education**			
Yes	196 (67.8%)	93 (32.2%)	0.002
No	288 (56.9%)	218 (43.1%)	

Chi-square test (χ^2^).

### Journals read by the dentists

Among the 484 dentists who affirmed having a habit of reading scientific journals, 63.8% responded they read either national or international journals. The dentists were requested to name the journals that they read, which generated about 925 journal citations. The citation rate of Brazilian (49.4%) and international journals (50.6%) was rather similar. The international journals most frequently mentioned were the *Journal of Periodontology* (2.7%), *Journal of Endodontics* (2.7%), *American Journal of Orthodontics and Dentofacial Orthopedics* (2.5%), and the *International Journal of Oral & Maxillofacial Implants* (2.3%).

## Discussion

This report is the first to address the use of scientific evidence in clinical practice among dentists working in Brazil. Although we could not determine the exact number of dentists that received the e-mail invitation to collaborate, the response rates for this type of study are usually low [19]. We used a self-administered questionnaire, which has the advantage of reaching a large sample size and covering a wide geographical area and population. Although the study findings might not be readily generalized to all dentists, the number of responses was similar or higher compared with studies carried out in other countries [9–11,13,15]. The study also reached all Brazilian territorial regions, although the North and Midwest regions accounted for only 4% of the respondents, perhaps because only five out of 11 regional councils from those regions agreed to collaborate, which can be suggestive of a geographical bias. However, the distribution of dentists working around the country is not uniform either; most dentists are concentrated in the Southeast, wherein most dental schools, graduate programs, and continuing education courses are concentrated [18]. A recent study indicated that the Southeast region of Brazil has three times more dentists than the recommendation of the World Health Organization [18].

Many respondents worked both in private practice and in the public sector, the latter of which is one of the major employers for dentists in Brazil considering that the public health system provides dental care [20]. However, working in the public sector was associated with a lower prevalence of the habit of reading journals and basing clinical decisions on the literature. Multiple factors potentially could be linked to these findings, such as the type of dental care provided in the public sector (usually primary care) [21], lack of time due to a potentially higher number of daily patient appointments, and the higher workload in the public sector compared with private practice [7]. These findings are of significant concern, given that most of the Brazilian population relies on public oral health care services [22]. The underlying reasons for these findings should be investigated in future studies.

A total of 76.3% of respondents reported dedicating their clinical practice to specialty areas, including even those who work in the public sector. The increasing number of specialization courses in health care is a trend in Brazil [23]. Many dentists who have completed postgraduate studies were enrolled in their second or third continuing education course. After completing postgraduate training, it is natural that the work of dentists would focus more on their postgraduate studies. In addition, 13.2% of respondents engaged in teaching. Dentists working in education are usually subjected to an environment of constant updating, thereby increasing their frequency of reading journals. In fact, engaging in teaching activities was associated with an increased habit of reading journals and practicing EBD. Dentists mentioned that clinical articles were the primary resource that led them to change their clinical practices or procedures. It seems that the dentists might pay more attention to articles that could directly assist their clinical practice, such as those that include images of clinical procedures or even step-by-step reports. Literature reviews were also frequently mentioned and might be effective in communicating evidence to dentists. However, systematic reviews were less often reported than narrative reviews. This is another point of concern because systematic reviews are in the top level of evidence pyramid when addressing data from randomized controlled trials, and are key to the practice of EBD.

The present findings indicate that there is room for improving EBD practice in Brazil. Overall, about 22.5% of dentists responded that they had never changed their clinical behavior based on scientific evidence. Using scientific evidence in the clinical practice is still a challenge for some dentists, although the reason for this has yet to be determined. One possible reason is that undergraduate dental schools, and even some advanced specialty training programs, might have a limited focus on the critical reading of scientific literature. When dentists are not used to reading articles, perhaps they could be more likely to follow their own intuition or experience when facing clinical challenges, or rely on advice from colleagues. Attending continuing education was associated with an increased practice of EBD, possibly a result of the discussions fomented during their educational activities. Another variable associated with the increased use of journals was holding an MSc or PhD. Obtaining these degrees involves heavy research and scientific writing, which naturally increases individuals’ exposure to journals and articles.

The population of the study was also associated with a higher frequency of reading scientific journals, probably due to the easier access to libraries and the higher number of universities and graduate courses in larger cities. Another information resource frequently mentioned was books, which accord with the findings of previous studies [10,14,15]. The dentists also reported a habit of consulting with colleagues on professional information, which is a behavior that seems common among health professionals [11]. Participation in conferences was also mentioned frequently, probably because this activity combines knowledge transfer and contact with colleagues [10]. Social media, blogs, and online courses, although still incipient resources, are potentially important for dental professional updating.

This study is the first to focus on the use of scientific information by dentists in Brazil. The practice of EBD was influenced by education and work variables of the dentists, thus the study hypothesis was accepted. Scientific journals were, notably, the second most frequent information resource mentioned. Thus, one might expect that dentists are practicing EBD on a large scale. In reality, however, it is believed that only a small portion of the dentists sent this questionnaire responded. It is natural to expect that those who opted to respond are more motivated to improve their skills and engage in knowledge updating, and are more interested in research than are those who opted not to respond. Therefore, this sample is likely more concerned with practicing EBD than are most dentists.

Dentistry is still a profession highly focused on clinical practice and personal and empirical experience; the incorporation of scientific evidence into this practice has been relatively slow. In that sense, translational research, which aims to translate research findings into health care practices, should be encouraged as a goal of researchers. EBD should also be part of training programs in dental schools in order to increase dentists’ confidence in using it in their practice. Fomenting the investigation of clinically relevant aspects in research should also be a continuing goal. Although some dentists might not find reading scientific articles enjoyable, all those involved in the process of making EBD a reality should understand the need for generating solid research evidence and their role in applying this evidence to the clinical environment.

## Conclusions

The present findings indicate that there is much room for improving the practice of EBD in Brazil, particularly among dentists in the public sector. Dentists who have changed clinical procedures based on prior literature tend to have done so after reading articles reporting on clinical outcomes. In addition, given that conferences and short-term courses were frequently mentioned as resources for professional updating, lecturers should understand their role in disseminating the practice of EBD among dentists.

## Supporting information

S1 File Original e-mail and questionnaire sent to the dentists. (PDF)

S2 File Underlying dataset. (XLSX)
