# Supplementary material for: Use of scientific evidence by dentists in Brazil: Room for improving the evidence-based practice
Source: PLoS One. 2018 Sep 19;13(9):e0203284. doi: 10.1371/journal.pone.0203284 (PMC6145584; doi:10.1371/journal.pone.0203284)
Supplement: S1 File — (PDF) [file pone.0203284.s001.pdf]

Caro membro do CRO,

Você está recebendo este e-mail por ser um cirurgião-dentista com cadastro ativo. A partir de agora você está sendo convidado a responder um questionário sobre o seu comportamento de atualização profissional e prática clínica com o objetivo de analisar o comportamento dos profissionais de odontologia do país quanto a busca de informação especializada.

Este questionário faz parte de uma dissertação de mestrado e somente os pesquisadores responsáveis terão acesso às suas respostas e os resultados serão disponibilizados em um periódico de acesso livre. Não haverá identificação dos entrevistados.

O tempo estimado para respondê-lo é de 5 minutos.

Em caso de dúvida não hesite em contatar os pesquisadores responsáveis:

Mestranda Ana Paula Rodrigues Gonçalves (anap.fo@ufpel.edu.br)
Prof. Dr. Rafael Ratto de Moraes (rafael.moraes@ufpel.edu.br)
Programa de Pós-Graduação em Odontologia, UFPel

Para acessar o questionário clique aqui

Desde já agradecemos sua atenção e colaboração,

Perfil informacional de Cirurgiões-Dentistas do Brasil

O questionário a seguir foi elaborado com o intuito de conhecer o comportamento de atualização e busca por informação especializada por cirurgiões-dentistas do Brasil.

Não há identificação do entrevistado. O resultado da pesquisa será divulgado em uma dissertação de mestrado e em um artigo científico a ser publicado em periódico científico de livre acesso.

O tempo estimado para responder as questões é de 5 minutos. As questões são, em geral, de múltipla escolha. Quando for possível assinalar mais de uma alternativa, esta informação estará presente no enunciado.

Ao final do questionário, por favor, não esqueça de clicar em “Enviar”. Agradecemos desde já sua participação!

Responsáveis pelo questionário:

Mestranda Ana Paula Rodrigues Gonçalves (anap.fo@ufpel.edu.br)
Prof. Dr. Rafael Ratto de Moraes (rafael.moraes@ufpel.edu.br)

Programa de Pós-Graduação em Odontologia, UFPel

*Obrigatório

1. **Caso concorde em participar da entrevista clique em "Aceito participar". Caso não deseje participar no momento, respeitaremos sua vontade! Se mudar de ideia, volte mais tarde.** *
Marcar apenas uma oval.

   ◯ Aceito participar.

Gênero

Por se tratar de uma pergunta pessoal, não é obrigatório respondê-la.

2. *Marcar apenas uma oval.*

   ◯ Feminino
   ◯ Masculino

Idade

3. **Selecione o ano de seu nascimento:** *
Marcar apenas uma oval.

   ◯ Após 2000
   ◯ 2000
   ◯ 1999
   ◯ 1998
   ◯ 1997

- 1996
- 1995
- 1994
- 1993
- 1992
- 1991
- 1990
- 1989
- 1988
- 1987
- 1986
- 1985
- 1984
- 1983
- 1982
- 1981
- 1980
- 1979
- 1978
- 1977
- 1976
- 1975
- 1974
- 1973
- 1972
- 1971
- 1970
- 1969
- 1968
- 1967
- 1966
- 1965
- 1964
- 1963
- 1962
- 1961
- 1960
- 1959

- 1958
- 1957
- 1956
- 1955
- 1954
- 1953
- 1952
- 1951
- 1950
- 1949
- 1948
- 1947
- 1946
- 1945
- 1944
- 1943
- 1942
- 1941
- 1940
- 1939
- 1938
- 1937
- 1936
- 1935
- 1934
- 1933
- 1932
- 1931
- 1929
- 1928
- 1927
- 1926
- 1925
- 1924
- 1923
- 1922
- 1921
- 1920

- 1919
- 1918
- 1917
- 1916
- 1915
- 1914
- 1913
- 1912
- 1911
- 1910
- 1909
- 1908
- 1907
- 1906
- 1905
- 1904
- 1903
- 1902
- 1901
- 1900

Em que estado você atua?

4. *

Marcar apenas uma oval.

- Acre - AC
- Alagoas - AL
- Amapá - AP
- Amazonas - AM
- Bahia - BA
- Ceará - CE
- Distrito Federal - DF
- Espírito Santo - ES
- Goiás - GO
- Maranhão - MA
- Mato Grosso - MT
- Mato Grosso do Sul - MS
- Minas Gerais - MG
- Pará - PA
- Paraíba - PB
- Paraná - PR
- Pernambuco - PE
- Piauí - PI
- Rio de Janeiro - RJ
- Rio Grande do Norte - RN
- Rio Grande do Sul - RS
- Rondônia - RO
- Roraima - RR
- Santa Catarina - SC
- São Paulo - SP
- Sergipe - SE
- Tocantins - TO

A cidade em que você atua é:

5. *

Marcar apenas uma oval.

- Capital
- Interior
- Atuo em ambos

Quantos habitantes possui a cidade em que você atua?

No caso de atuar em mais de uma cidade, responda sobre aquela com maior número de habitantes.

6. *

Marcar apenas uma oval.

- Até 5 mil habitantes
- Entre 5 e 10 mil habitantes
- Entre 10 e 50 mil habitantes
- Entre 50 e 100 mil habitantes
- Entre 100 e 300 mil habitantes
- Entre 300 e 500 mil habitantes
- Entre 500 e 700 mil habitantes
- Entre 700 mil e 1 milhão de habitantes
- Acima de 1 milhão de habitantes

Considerando o ano em que você concluiu o curso de graduação em Odontologia, há quantos anos você exerce a profissão?

7. *

Marcar apenas uma oval.

- 1
- 2
- 3
- 4
- 5
- 6
- 7
- 8
- 9
- 10
- 11
- 12
- 13
- 14
- 15
- 16
- 17
- 18
- 19
- 20

- 21
- 22
- 23
- 24
- 25
- 26
- 27
- 28
- 29
- 30
- 31
- 32
- 33
- 34
- 35
- 36
- 37
- 38
- 39
- 40
- Mais de 40

Em que estado está localizada a Faculdade de Odontologia em que você se graduou?

8. *

Marcar apenas uma oval.

- Acre - AC *Ir para a pergunta 9.*
- Alagoas - AL *Ir para a pergunta 9.*
- Amapá - AP *Ir para a pergunta 9.*
- Amazonas - AM *Ir para a pergunta 9.*
- Bahia - BA *Ir para a pergunta 9.*
- Ceará - CE *Ir para a pergunta 9.*
- Distrito Federal - DF *Ir para a pergunta 9.*
- Espírito Santo - ES *Ir para a pergunta 9.*
- Goiás - GO *Ir para a pergunta 9.*
- Maranhão - MA *Ir para a pergunta 9.*
- Mato Grosso - MT *Ir para a pergunta 9.*
- Mato Grosso do Sul - MS *Ir para a pergunta 9.*
- Minas Gerais - MG *Ir para a pergunta 9.*
- Pará - PA *Ir para a pergunta 9.*
- Paraíba - PB *Ir para a pergunta 9.*
- Paraná - PR *Ir para a pergunta 9.*
- Pernambuco - PE *Ir para a pergunta 9.*
- Piauí - PI *Ir para a pergunta 9.*
- Rio de Janeiro - RJ *Ir para a pergunta 9.*
- Rio Grande do Norte - RN *Ir para a pergunta 9.*
- Rio Grande do Sul - RS *Ir para a pergunta 9.*
- Rondônia - RO *Ir para a pergunta 9.*
- Roraima - RR *Ir para a pergunta 9.*
- Santa Catarina - SC *Ir para a pergunta 9.*
- São Paulo - SP *Ir para a pergunta 9.*
- Sergipe - SE *Ir para a pergunta 9.*
- Tocantins - TO *Ir para a pergunta 9.*
- Não me graduei no Brasil *Ir para a pergunta 10.*

Em que cidade?

9. *

Ir para a pergunta 11.

Você respondeu que não se graduou no Brasil. Em que país foi sua graduação?

10. *

Qual sua titulação máxima?

11. *

Marcar apenas uma oval.

- Graduação
- Atualização/Capacitação/Aperfeiçoamento
- Especialização
- Residência
- Mestrado Profissional
- Mestrado Acadêmico
- Doutorado
- Pós-Doutorado
- Outro: ______________________

Qual sua área predominante de atuação?

Mesmo que você atue em mais de uma área, selecione aquela na qual você emprega a maior parte do seu tempo clínico.

12. *

Marcar apenas uma oval.

- Dentística
- Disfunção Têmporo-mandibular e Dor Orofacial
- Prótese Dentária
- Prótese Buco-maxilo-facial
- Cirurgia e Traumatologia Buco-maxilo-facial
- Implantodontia
- Periodontia
- Endodontia
- Ortodontia
- Estomatologia
- Patologia Bucal
- Radiologia
- Saúde Coletiva
- Odontogeriatria
- Odontopediatria
- Odontologia para Pacientes com Necessidades Especiais
- Odontologia do Trabalho
- Odontologia Legal
- Clínica Geral
- Outro: ____________________

Em que tipo de serviço você atua?

Marque mais de uma alternativa, se for o caso.

13. *

Marque todas que se aplicam.

- [ ] Posto de saúde ou outro serviço público similar
- [ ] Consultório odontológico
- [ ] Clínica odontológica privada
- [ ] Serviço público mas não diretamente com atividade clínica
- [ ] Serviço privado mas não diretamente com atividade clínica
- [ ] Instituição de ensino pública
- [ ] Instituição de ensino privada
- [ ] Outro: ____________________

Você acha importante que o Cirurgião-Dentista frequente cursos visando à educação continuada?

Educação continuada: qualquer tipo de aprendizagem após a graduação.

14. *

Marcar apenas uma oval.

- Sim
- Não

Você atualmente cursa pós-graduação?

15. *

Marcar apenas uma oval.

- Sim *Ir para a pergunta 16.*
- Não *Ir para a pergunta 18.*

Que tipo de pós-graduação você cursa no momento?

16. *

Marcar apenas uma oval.

- Atualização/Capacitação/Aperfeiçoamento *Ir para a pergunta 17.*
- Especialização *Ir para a pergunta 17.*
- Residência *Ir para a pergunta 17.*
- Mestrado Acadêmico *Ir para a pergunta 17.*
- Mestrado Profissional *Ir para a pergunta 17.*
- Doutorado *Ir para a pergunta 17.*
- Pós-doutorado *Ir para a pergunta 17.*
- Outro: ______________________________

Esta é a sua primeira pós-graduação?

17. *

Marcar apenas uma oval.

- Sim *Ir para a pergunta 19.*
- Não *Ir para a pergunta 18.*

Que tipo de pós-graduação você já cursou?

Marque mais de uma alternativa, se for o caso.

18. *

Marque todas que se aplicam.

- [ ] Atualização/Capacitação/Aperfeiçoamento
- [ ] Especialização
- [ ] Residência
- [ ] Mestrado Acadêmico
- [ ] Mestrado Profissional
- [ ] Doutorado
- [ ] Pós-doutorado
- [ ] Não cursei
- [ ] Outro: ______________________

Você é ou já foi docente ou responsável por atividades de ensino?

19. *

Marcar apenas uma oval.

- ( ) Sim *Ir para a pergunta 20.*
- ( ) Não *Ir para a pergunta 21.*

De que tipo?

Marque mais de uma alternativa, se for o caso.

20. *

Marque todas que se aplicam.

- [ ] Atividades de Graduação
- [ ] Atividades de Atualização/Capacitação/Aperfeiçoamento
- [ ] Atividades de Especialização
- [ ] Atividades de Mestrado Profissional
- [ ] Atividades de Pós-graduação Stricto Sensu
- [ ] Outro: ______________________

Em uma escala de 0 a 10, assinale o seu conhecimento sobre os seguintes termos:

21. **"Odontologia baseada em evidências"** *
Marcar apenas uma oval.

	0	1	2	3	4	5	6	7	8	9	10	
Nunca ouvi falar												Conheço bem o termo

22. **"Evidência científica"** *
Marcar apenas uma oval.

	0	1	2	3	4	5	6	7	8	9	10	
Nunca ouvi falar												Conheço bem o termo

23. **"Pesquisa clínica"** *
Marcar apenas uma oval.

	0	1	2	3	4	5	6	7	8	9	10	
Nunca ouvi falar												Conheço bem o termo

24. **"Pesquisa epidemiológica"** *
Marcar apenas uma oval.

	0	1	2	3	4	5	6	7	8	9	10	
Nunca ouvi falar												Conheço bem o termo

25. **"Periódico científico"** *
Marcar apenas uma oval.

	0	1	2	3	4	5	6	7	8	9	10	
Nunca ouvi falar												Conheço bem o termo

Quando você sente necessidade de ler ou se atualizar sobre um tema odontológico, onde você busca informações?

Marque mais de uma alternativa, se for o caso.

26. *

Marque todas que se aplicam.

- [ ] Não sinto necessidade
- [ ] Com colegas
- [ ] Congressos
- [ ] Cursos de curta duração
- [ ] Cursos de longa duração
- [ ] Cursos on-line
- [ ] Blogs
- [ ] Redes sociais
- [ ] Sites de instituições de ensino ou pesquisa
- [ ] Livros
- [ ] Periódicos científicos
- [ ] Revistas clínicas
- [ ] Jornais de Conselhos de Classe (ex: CRO, CFO)
- [ ] Jornais de Associações de Classe (ex: ABO, ABCD)
- [ ] Outro: ____________________

Com que frequência você busca informações para se atualizar sobre odontologia?

27. *

Marcar apenas uma oval.

- ( ) Não busco informação *Ir para a pergunta 28.*
- ( ) Diária
- ( ) Semanal *Ir para a pergunta 28.*
- ( ) Mensal *Ir para a pergunta 28.*
- ( ) Outro: ____________________

Você tem o costume de acessar informações em periódicos científicos?

Periódicos científicos: revistas que divulgam informações advindas de pesquisas científicas e revisadas criteriosamente garantir qualidade e validade da publicação.

28. *

Marcar apenas uma oval.

- ( ) Sim *Ir para a pergunta 29.*
- ( ) Não *Ir para a pergunta 34.*

Com que frequência?

29. *

Marcar apenas uma oval.

- Diária
- Semanal
- Mensal
- Outro: ______________________

Esses periódicos são:

Marque mais de uma alternativa, se for o caso.

30. *

Marque todas que se aplicam.

- [ ] Nacionais
- [ ] Internacionais

Quais periódicos?

Cite os periódicos abaixo, separados por vírgulas (no máximo 5).

31. *

____
____
____
____
____

Que tipo de artigo você costuma ler em periódicos científicos?

Marque mais de uma alternativa, se for o caso.

32. *

Marque todas que se aplicam.

- [ ] Relato de caso
- [ ] Opinião de especialistas
- [ ] Editoriais
- [ ] Pesquisa laboratorial
- [ ] Pesquisa clínica
- [ ] Pesquisa epidemiológica
- [ ] Revisão de literatura
- [ ] Revisão sistemática ou metanálise
- [ ] Outro: ______________________

Com que frequência você avalia que artigos científicos não condizem com a prática clínica?

33. *

Marcar apenas uma oval.

	0	1	2	3	4	5	6	7	8	9	10	
Nunca	◯	◯	◯	◯	◯	◯	◯	◯	◯	◯	◯	Sempre

Você já alterou algum procedimento ou conduta clínica com base em informações que obteve em um periódico cientifico?

34. *

Marcar apenas uma oval.

- ◯ Sim
- ◯ Não *Ir para a pergunta 36.*

Que tipo de artigo fez você adotar uma nova conduta?

35. *

Marque todas que se aplicam.

- ☐ Relato de caso
- ☐ Opinião de especialistas
- ☐ Editoriais
- ☐ Pesquisa laboratorial
- ☐ Pesquisa clínica
- ☐ Revisão de literatura
- ☐ Revisão sistemática ou metanálise
- ☐ Outro: ____________________

Ir para a pergunta 37.

Por que você não alterou condutas clínicas com base em informações veiculadas em periódicos científicos?

36. *

Marcar apenas uma oval.

- ( ) Não leio periódicos científicos *Ir para a pergunta 37.*
- ( ) Não acredito nas informações veiculadas em periódicos científicos *Ir para a pergunta 37.*
- ( ) Acho que os artigos de periódicos científicos não refletem a realidade clínica *Ir para a pergunta 37.*
- ( ) As informações importantes são veiculadas em inglês e não tenho domínio desse idioma *Ir para a pergunta 37.*
- ( ) Outro: ____________

Indique o quanto sua tomada de decisões clínicas se baseia em artigos científicos:

37. *

Marcar apenas uma oval.

	0	1	2	3	4	5	6	7	8	9	10	
Nenhuma influência	( )	( )	( )	( )	( )	( )	( )	( )	( )	( )	( )	Muita influência

Powered by
Google Forms
